# Examining universal access to acute hospital care in Ireland during the first three months of COVID-19: Lessons from the policy process

**DOI:** 10.12688/hrbopenres.13848.1

**Published:** 2024-02-15

**Authors:** Luisne Mac Conghail, Sarah Parker, Sara Burke

**Affiliations:** 1 Centre for Health Policy and Management Discipline of Public Health and Primary Care, School of Medicine, The University of Dublin Trinity College, 2-4 Foster Place, Dublin, 2, Ireland

**Keywords:** Ireland, health system, health reform, COVID-19, health policy, universal healthcare, public hospitals, private hospitals

## Abstract

**Background:**

The onset of the COVID-19 pandemic prompted the Irish state to address unprecedented challenges by prioritising public health and equitable access to care. Confronted with the threat of overwhelmed capacity in acute public hospitals, Ireland, initiated a historic Safety Net Agreement (SNA) with 19 private hospitals in March 2020, marking the inaugural establishment of universal single-tier access to hospital care in Ireland. This research aimed to comprehensively examine the policy process underlying the agreement, deriving insights for the implementation of Universal Healthcare.

**Methods:**

Employing a retrospective qualitative case study approach, the research examined the policy process, including the content, context, actors, and mechanisms involved in the SNA’s implementation. The study used a dataset of 68 policy documents and conducted seven key informant interviews.

**Results:**

Responding to the pandemic, Ireland classified COVID-19 as a notifiable infectious disease under the 1947 Health Act, exempting affected patients from public hospital charges. The government swiftly implemented health policy measures for universal access through the SNA, recognising challenges in public healthcare capacity and ethical dilemmas within the two-tier hospital system. The agreement's discontinuation was heavily influenced by private hospital consultants, revealing strained relationships and misunderstandings of the private sector. The ongoing policy drift since the pandemic highlights the need for a reassessment of private-sector strategies to alleviate capacity pressures in Ireland's public health system. The SNA also sparked the consideration of a Universal Health Insurance model for Ireland's hospital care.

**Conclusions:**

Exploring the policy dynamics at the intersection of public and private healthcare, the study imparts lessons for health system reform. The insights have the potential to contribute to long-term goal alignment, robust governance practices, and trust-building mechanisms for effective public-private collaborations in a two-tier health system, offering valuable guidance for future healthcare policy and implementation.

## Introduction

The COVID-19 pandemic presented an unprecedented global health crisis, laying bare deficiencies in social protection and structural inequalities ^1^. European states and their health systems found themselves confronted with heightened demands for swift policy action to mitigate the spread of infection and safeguard public health, which required a comprehensive reconfiguration of healthcare systems ^2^. The pandemic had a significant impact on local strategies, as it considered various attributes of healthcare systems, including their structural facets, the presence and influence of the private hospital sector, and the availability and occupancy rates of acute hospital beds ^2– 6^. In response, adopted plans included ensuring sufficient physical infrastructure, securing extra essential equipment, and expanding and reorganising the healthcare workforce to effectively manage the surge in demand from COVID-19 ^2, 5, 7, 8^.

Although countries' responses to the pandemic showed varying levels of sustained effectiveness, research suggests that resilient health systems capable of effectively harnessing health surveillance with the fundamental pillars of Universal Healthcare (UHC) were often better prepared to safeguard their populations against the pandemic ^9– 13^. As a set of health policy goals, UHC depends on ensuring access to comprehensive, appropriate, timely, and quality health services without imposing a financial burden ^14^. The pandemic has underscored a renewed significance on universal access to healthcare, proving that when the healthcare needs of a portion of the population are unattended, the whole population is at risk ^12^. In response, some governments devised and implemented various UHC policy measures during COVID-19, emphasising enhanced health service accessibility and responsiveness for their populations ^15– 18^.

Several European countries, including Ireland, England, Italy and Greece, utilised and integrated private hospitals into the public system response to ensure access to all hospital care for COVID-19 patients and critical non-COVID elective procedures ^3, 19^. It is essential to distinguish that the engagements during the COVID-19 pandemic differed significantly from traditional public-private partnerships, usually associated with high-cost and long-term projects. Instead, private sector involvement in the pandemic response was characterised by urgent needs, limited supplies, unconventional methods, and notably shortened timelines compared to non-emergency situations ^19^. In the context of UHC, the WHO defines private sector engagement as the meaningful inclusion of private providers for service delivery in mixed health systems ^20^. This requires a comprehensive approach to the governance of health systems, spanning both private and public sectors. The focus is on defining appropriate areas for private sector engagement and implementing governance strategies that align with the health system's goals, ensuring access to high-quality care and financial protection for patients, regardless of where they seek treatment ^19– 21^.

Effective private-sector engagement mandates aligning private-sector initiatives with public-sector health goals, demanding a commitment to actively support the government's agenda ^19, 20^. Previous research has investigated the involvement of the private sector in service delivery during the COVID-19 pandemic ^19^, as well as the unique challenges associated with implementing universal healthcare in systems marked by a prominent two-tier public/private system ^22– 25^. In addition, the literature underscores the significance of insights derived from private sector engagement during the COVID-19 emergency period, emphasising the importance of these learnings. This is particularly crucial considering the potential evolution of emergency policies as enduring features of health systems ^26, 27^.

Against this backdrop, it's crucial to understand how universalist COVID-19 policies aimed at increasing public healthcare provision intersect and play out in healthcare systems with a strong private sector presence. This intersection gains heightened relevance within the Irish context, where the interplay between universalist COVID-19 policies and a substantial private sector holds particular significance. The emergence of UHC principles played a central role in responding to the COVID-19 crisis ^17, 18, 28, 29^. Although additional capacity from the private sector was deemed necessary, the primary emphasis remained on upholding an equitable public sector-driven pandemic response ^30^. This positioning emphasises the study's contribution to unravelling the complexities of navigating universalist policies in the presence of a strong private sector within the Irish healthcare landscape.

## Background

In March 2020, the Irish public health system - the Health Service Executive (HSE) and the Department of Health, concerned with the threat COVID-19 posed on existing capacity constraints in acute public hospitals, reached an agreement with the Private Hospital Association (PHA) entitled the Safety Net Agreement (SNA). This enabled the HSE to have sole control over private hospital capacity (19 private hospitals) for all Irish people from April to June 2020.

The Irish health system comprises a complex mixture of public and private financing and delivery. In 2019, pre-pandemic, the system was financed by general taxation (74%), voluntary health insurance (14%), and out-of-pocket payments (12%) ^31^. There are 51 publicly funded hospitals in Ireland. The acute hospital sector comprises of three models: i) 32 public entities governed and run directly by the state; ii) 19 independently owned voluntary bodies funded by the state under Section 38 of the 2004 Health Act governed by non-profits, seven of which are owned by faith-based organisations with a further five having some degree of faith-based involvement in their governance arrangements; and iii) 19 private for-profit organisations ^32– 36^. It was the 19 private for-profit hospitals which were the focus of the SNA.

Ireland's public healthcare system does not offer a comprehensive universal entitlement to healthcare, underscoring the intricate complexity of the system ^32^. Eligibility and income mean testing exist for services specified under the 1970 Health Act. This divides Irish citizens into two categories: Category 1, individuals with full eligibility who qualify for the General Medical Card Scheme (31.8% of the population in 2019) based on means-testing receive free access to primary and acute care, and co-payments for prescription medicine ^37^. Category 2, individuals with limited eligibility pay out-of-pocket costs at full price or co-payment ^32^. Some individuals are eligible for a GP visit card that allows patients under six years old and 70+ to access GP care without charge ^38^. Individuals whose income falls below a specific threshold (yet remains above the Category 1 income threshold) are also eligible for a GP visit card. In addition, in 2020, public hospital care included a €100 fee for emergency department visits without admission, except for individuals with a general practitioner's referral or those holding a medical card. There was a nightly charge of €80 for inpatient treatment, with an annual cap of up to €800 ^32, 38^.

UHC in Ireland is a contentious issue marked by power struggles and opposition from political and economic elites, religious institutions, and certain medical professionals ^34, 39, 40^. Despite gaining national independence in 1922, Irish public policy remained under the religious institutions powerful influence, which dominated key societal domains, including education and healthcare ^34, 40^. During this time, the nation heavily depended on health services established and governed by religious orders, funded through a combination of charitable endowments, parliamentary grants, and a national lottery ^34, 40, 41^. However, a pivotal transformation has unfolded since the mid-1980s as the influence of the Catholic Church on Irish society has continuously waned. During this period, a significant paradigm shift emerged, with Irish political parties embracing market-driven solutions for the development of the healthcare system, reflecting the prevailing public policy trends seen in both the UK and the USA ^34, 41, 42^.

Over the past four decades, the promotion of market-based ideologies and neoliberal policies such as austerity-driven spending reductions and privatisation has been the driving force behind the substantial growth of the Irish private acute hospital sector, further complicating an already underdeveloped and under-resourced public healthcare system ^34, 43– 48^. As a consequence, the Irish healthcare system stands fragmented, marked by capacity constraints, a lack of universal entitlements, and long waiting times for essential care ^23, 43, 45, 46, 49, 50^. The entitlement to the public health system becomes more theoretical than practical ^39^. Consequently, patients with adequate financial resources are incentivised to purchase private health insurance (46% of the population in 2019), providing faster access to care across the health system, including some public facilities ^51^. As a result, significant disparities in health outcomes have emerged in Ireland, with vulnerable population groups experiencing disproportionate impacts in terms of poor access and quality ^48, 52– 54^.

In 2011, a political commitment was made for the first time to introduce UHC through the introduction of Universal Health Insurance (UHI) as a mechanism to end the two-tier hospital system in Ireland. Subsequently, a white paper detailing these intentions was published by the government in 2014 but was abandoned in 2015 on cost grounds ^55, 56^. Nevertheless, UHC remained on the political agenda ^57, 58^. Since 2017, there has been cross-party political support for 'Sláintecare' – Ireland’s 10-year national health policy, which is underpinned by a commitment to UHC and aims to establish a single-tier health system that provides timely access to quality care solely based on health need ^59, 60^. However, implementation is slow ^61^, supporting the view that there is an absence of clarity and agreement on definitions of UHC in Ireland, including universal entitlement to care and the mechanisms to achieve this ^50, 62^.

Previous research has emphasised the significance of the SNA in Irish health policy, particularly its role in establishing a unified single-tier public acute hospital system – an unprecedented development for Ireland, albeit for a 3-month period ^18, 28, 63^. However, a knowledge gap remains regarding the specific mechanisms used to achieve this, along with the experiences and insights of stakeholders in the negotiation and delivery of the SNA. Addressing this gap presents a critical opportunity to better understand the policy process and to harness vital policy insights into public-private relationships in the healthcare sector that can guide future health reform efforts towards achieving UHC in Ireland.

## Research aim

This research aims to comprehensively examine the SNA established between the HSE and Irish private hospitals in March 2020 as a case study within the Irish health system, where a UHC policy (within the acute hospital sector) was implemented in response to the COVID-19 pandemic. The primary objective of this research is to analyse all parts of the policy process, including the substantive content of the SNA, its contextual background, the roles played by various actors involved, and the mechanisms employed to enact this policy response. Through this investigation, valuable insights are derived from this specific policy change, offering a deeper understanding of UHC implementation in two-tier public/private health systems.

## Methods

This study forms one of the workstreams within the Health Research Board (HRB)-funded Foundations’ research project that aims to harness key learnings from Ireland’s health system response to COVID-19 with a view to informing the implementation of Ireland’s ten-year health reform plan, Sláintecare ^17, 29, 38, 64– 66^. 

The research employs a retrospective qualitative case study approach to explore the policy process regarding the agreement with Irish private hospitals to access capacity during the Covid-19 pandemic from March–May 2020. The research design incorporates two qualitative methods of: documentary analysis and semi-structured interviews.

The intricate nature of health policy and system responses during a crisis necessitates a qualitative approach, as qualitative research facilitates a comprehensive exploration of decision-making processes, stakeholder interactions, and contextual nuances crucial for capturing the multifaceted dynamics that shape policy outcomes ^67– 69^. This approach is particularly relevant in Health Policy and Systems Research (HPSR), which aims to provide explanatory insights that inform health system improvements and outcomes ^70^.

Within qualitative research, case study methodology plays a central role and enables a detailed examination of specific health policy instances or system features ^71, 72^. These in-depth investigations involve analysing formal documents, guidelines, and the actions of key stakeholders to describe and explain real-life phenomena of interest ^73^.

Two fundamental qualitative methods, documentary analysis and semi-structured interviews, are integrated to enhance the research's robustness. While documentary analysis offers a structured understanding of textual content, it may lack the full operational context ^68^. Semi-structured interviews complement this by providing full contextual depth and a broader perspective, thus bridging the gap between documented narratives and actual practice ^74^.

### Data collection

This study encompassed documentary analysis of 68 documents related to the SNA whilst simultaneously conducting qualitative interviews with 7 senior managers, policymakers and clinicians drawn from the public (n=5) and private sectors (n=2). These participants possessed direct experience in negotiating and/or executing the SNA. Data collection for the document analysis involved a comprehensive review of public and academic literature pertaining to the agreement spanning May 2017 to August 2020. These are listed in Supplementary File 1. A detailed overview of the dataset, including the quantity and nature of included documents, is presented in Table 1.

**Table 1.** Detailed Overview of Dataset.

Document Type	Number (N)
Health Service Documents	19
Media Reports and Press Releases	16
Policy Documents and Strategies	11
Academic and Grey Literature	10
Parliamentary Correspondence - Committees, Debates and Questions	6
Commissioned Reports	3
Legislation	3
**Total**	**N= 68**

May 2017 was selected as the proposed start date due to its significance as Ireland's first government health policy commitment to UHC. It provides a further contextual understanding of the policy landscape surrounding the agreement. Additionally, historically relevant documents such as the 1947 and 1970 Health Acts, which establish the legal basis for current entitlements to healthcare and infectious disease management, were included. Databases searched included government websites such as Gov.ie and the Department of Health webpage, Oireachtas website, Lenus (the Health Research Repository of the Irish Health Service), and LexisNexis. Additional documents were obtained from the respondents if they were not accessible in the public domain, which provided the research with a greater understanding of the SNA than was previously and publicly available.

A purposive snowball sampling approach was employed for the qualitative interviews to select participants from four distinct clusters of policy actors: government officials, healthcare managers, professional associations, and healthcare professionals. Following ethical approval from the Research Ethics Committee of the Centre for Health Policy and Management and Centre for Global Health in Trinity College Dublin's School of Medicine in 2021, prospective respondents who met the criteria were invited via email to participate in semi-structured interviews. A total of 24 participants were invited, with 7 agreeing to be interviewed. The interviews took place online via online conferencing and in person at a secure location between May and June 2021 and lasted approximately 40-75 minutes each. Two sets of topic guides, tailored for public sector and private sector actors, were used during the interviews. The questions asked are available in the extended data file. The topics explored included:

- The policy goals, impetus, and problem definition, including what the agreement aimed to address and the concerns.
- The methods and boundaries established to support this temporary collaboration.
- The primary groups of stakeholders and their contributions in negotiating and implementing the deal.
- The process, key challenges and areas involved in formulating, implementing, and evaluating the SNA.
- The impacts of this agreement on healthcare reform in Ireland.

With participants’ consent, all interviews were digitally recorded and transcribed verbatim using otter.ai.

### Data Analysis

The study adopted an inductive qualitative approach for data analysis, guided by Braun and Clarke's thematic analysis ^75^. Word processing and spreadsheet software (Microsoft Word and Excel) were utilised to organise the collected data systematically. Transcripts and documents underwent initial coding for themes primarily by one researcher (LMC). A second researcher (SB) coded 30% of the same. Data triangulation from interviews and documentary analysis was methodically incorporated throughout the data analysis process, bolstering the findings' trustworthiness, reliability, and validity ^76^. Extensive discussions within the research team led to the development of coding categories that emerged from the data *a priori* ^77^. Where there was inconsistency in understandings and codes, this was discussed for clarification until consistency in coding was achieved. This back-and-forth process of interviews, document analysis validation, and discussion with the research team facilitated a thorough and robust data analysis, yielding valuable insights for health system reform.

### Strengths and limitations

This study adopts a mixed-method qualitative approach, combining interviews and documentary analysis, to deeply investigate a specific policy process. The use of formal documents and stakeholder interviews provides a detailed understanding of the COVID-19 policy response and its implications for Universal Health Coverage (UHC) in Ireland.

However, the study has limitations, including a restricted number of interviews, especially from the private sector. Out of fifteen targeted private sector representatives, only two participated. The small participant pool reflects the study's unique context, involving a limited number of key individuals from both sectors.

The research, based solely on Irish data, urges caution in generalising findings to other contexts due to different circumstances. Nonetheless, it lays groundwork for further research, suggesting the value of comparative studies across Europe to understand COVID-19 policy variations. Such studies can enrich health policy decision-making in Ireland and internationally.

## Findings

**Figure 1.**
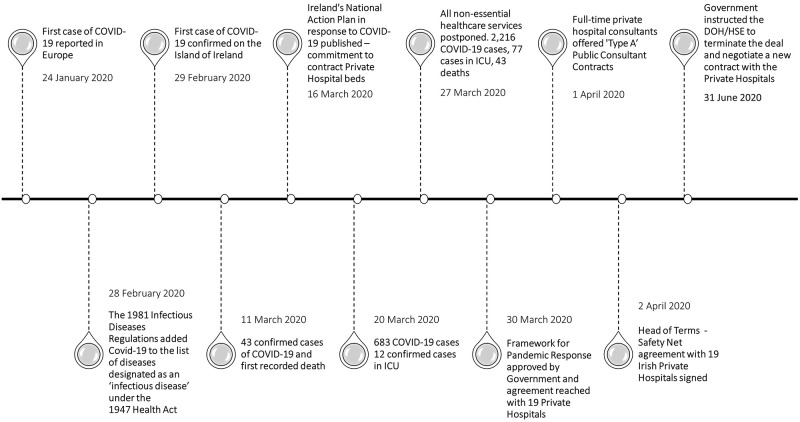
Timeline of Key Events ^30, 77– 89^.

The findings below are presented as:

1. A narrative timeline of the SNA ( Figure 1) ^30, 77– 89^, its context, content, and process, i.e., how it was implemented
2. The two prominent themes that emerged:
  a. The influence of the medical profession
  b. Public administrative capacity to engage the private sector.

### Narrative timeline

In the initial quarter of 2020, as the COVID-19 pandemic unfolded in Europe, the Irish health system faced an unprecedented challenge in rapidly expanding its capacity to accommodate the increasing number of patients ^18^. At the outset, Ireland grappled with a constrained supply of acute hospital beds and consistently high occupancy rates, with 250 critical care beds and 11,000 inpatient beds, which operated near 100% occupancy ^40, 41^. Anticipating a surge in illness presenting at hospitals, the exhaustion of Ireland's limited ICU capacity coupled with a heightened awareness of Europe's evolving public health situation, health authorities and the state promptly launched a public health response. 

The first and most crucial element of the state and health system approach to confronting the COVID-19 pandemic was on 28 February, the inclusion of COVID-19 in the list of notifiable infectious diseases stipulated in the Infectious Disease Regulation 1981 of the 1947 Health Act ^80^. This legislative provision was explicitly crafted to contain the spread of infectious diseases and exempt patients from statutory public hospital charges.

On 16 March, the government published Ireland’s National Action Plan in Response to COVID-19 ^30^, delineating strategies for ensuring sufficient access to hospital care for the public. The plan outlined measures that the HSE and the Department of Health would undertake to bolster bed and hospital capacity, including forging partnerships with the private hospital sector. From the outset, there was substantial support for collaborating with the private sector. What became evident during the qualitative interviews was that this support stemmed from the determination of officials and politicians to increase capacity to ensure universal and equitable access to COVID-19 care, aligning with the mantra of the Infectious Disease Regulation and existing policy objectives of 'Sláintecare' ^59^. This approach was encapsulated in ‘we’re all in this together’ which was often cited during interviews and resonated throughout the media during the initial stages of the pandemic ^81, 82^.

**Figure 2.**
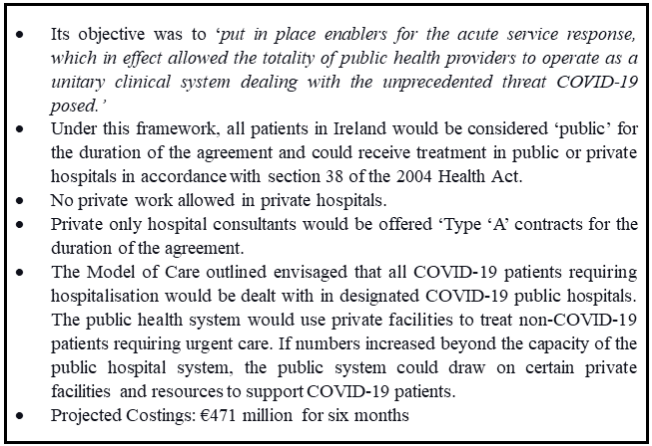
Details of ‘Framework of Pandemic Response’ ^83^.

In response to the government's mandate as outlined in Ireland’s National Action Plan, the HSE and the Department of Health engaged in an intensive three-week negotiation process with the Private Hospital Association (PHA), the representative body for the private hospital sector in Ireland. The central objective of these negotiations was to secure the utilisation of private hospital facilities for Ireland's public health system in anticipation of the surge of COVID-19 cases. Once negotiations were finalised, public health officials presented a 'Framework for Pandemic Response' to the government for approval, outlining the workings of the collaborative engagement between the HSE and private hospitals to secure additional hospital capacity (see Figure 2) ^83^. On March 30, 2020, the government granted its approval ^84^. Under this framework, all patients in Ireland would be considered ‘public’ for the duration of the agreement, granting them access to treatment in both public and private hospitals ^83– 85^. Furthermore, the public health system would gain access to an additional 2,500 private hospital beds, including over 100 critical care beds and 200 ventilators ^79^.

**Figure 3.**
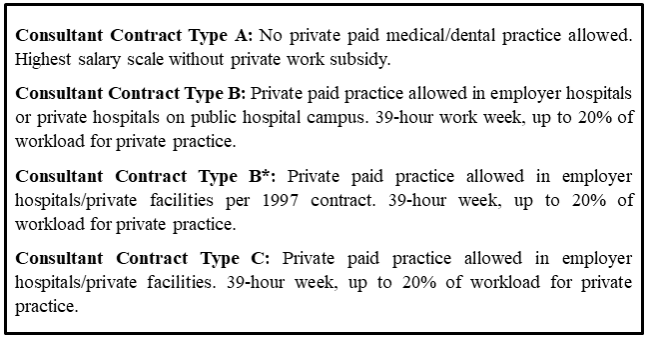
Summary of consultant contracts available in the Irish public health system in 2020 ^95^.

**Figure 4.**
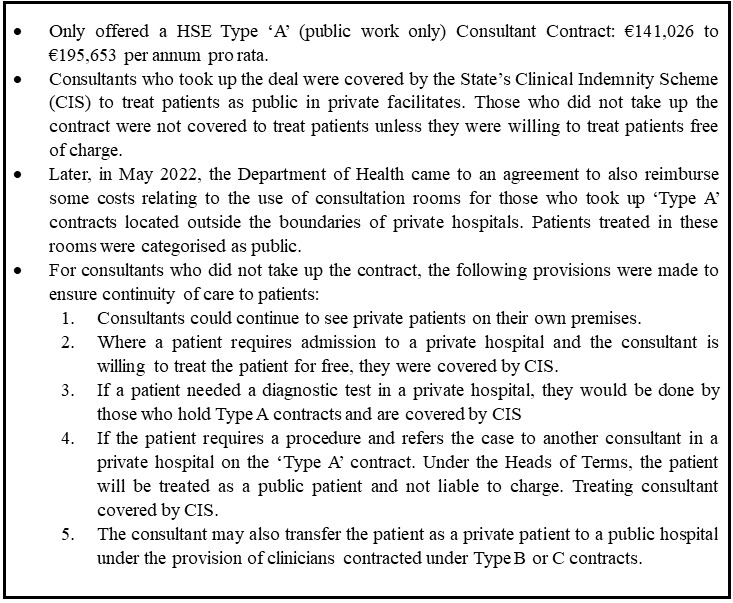
Details of the contract offered to Private Hospital Consultants under the Safety Net Deal ^80– 83, 94, 95^.

Most consultants in Irish private hospitals operate as independent contractors. There are some specific instances where they are employed directly by the private entity. In the Framework for Pandemic Response, it was decided that the state would offer existing private-only consultants (who would have had no state contract) a 'Type A' contracts for the duration of the SNA (see Figure 3), allowing them to participate in public healthcare work exclusively ^84– 88^ (see Figure 4).

Following the government's approval, on 1 April, the HSE offered 'Type A' contracts to consultants who only work in private hospitals ^88^. Heads of Terms (HoT) were drafted per the previously established 'Framework for Pandemic Response' document ^84^. Nineteen private hospitals signed the HoT on 2 April 2020 ^84^. Consequently, the public health system gained access to the entire capacity of the private hospital system for an initial three-month period, with a provision to extend the arrangement as required. No private work was permitted in any private hospital during this time.

Simultaneously, the government made a critical decision following a recommendation from the National Public Health Emergency Team (NPHET) on 27 March to postpone all non-essential healthcare services across the public system ^89^. The decision was made to safeguard capacity in public hospitals and mitigate the risk of infection transmission within hospital premises by reducing patient attendance ^38, 90– 92^. During this period, the available capacity in the private hospitals was used to support the HSE in delivering urgent and time-dependent treatments and procedures, particularly cancer treatment, cardiovascular surgeries, transplant surgery, chemotherapy, radiological tests, and procedures ^84^. Moreover, the private hospital capacity remained readily accessible as the need arose, aligning closely with the original primary objective of the arrangement ^85^. However, it is also noteworthy that the suspension of non-essential care, accompanied by the 'buy out' agreement, prevented substantial financial losses for the private sector. This mutually beneficial arrangement offered the private sector a stable income during a highly uncertain period when their business model was particularly vulnerable.

As the rising cost of the SNA began to emerge and the COVID-19 surge expected and planned for never materialised, the political consensus supporting the deal started to wane, leading to public, political and media commentary on the agreement’s efficacy ^63, 79, 92– 97^. Towards the end of the agreement, in June 2020, it was suggested that the public system did not utilise the full available capacity provided for under the agreement and that, at €300 million for three months (versus €471 million for six months initially put to government) the deal was not providing value for money ^79^.

Stakeholders involved in the agreement’s negotiation and delivery shared government views that the private hospital arrangement was not used to maximum effect ^98^. Then Secretary-General of the Department of Health, Jim Breslin, responded to the criticisms of capacity under-utilisation stating, *'A fire alarm went off, and we sent four fire tenders to put out the fire, and people are now saying we should have sent only three. I am very happy that we sent four fire tenders because what if it needed more than three’* ^79^.

After three months, on 30 June 2020, the government instructed the HSE to terminate the existing HoT and, together with the Department of Health and the Department of Public Expenditure and Reform, to negotiate a new contract with the private hospitals to provide additional public capacity ^92^.

### The influence of the medical profession

In Ireland, medical consultants, in their private capacity, typically operate as independent contractors for private hospitals. They are not usually employed directly by hospitals but provide their medical services independently under private practice agreements in consulting rooms often off-site and providing medical care in private hospitals. A small group of these private hospital consultants working exclusively in the private sector raised initial criticisms and objections to the SNA. Media reports highlighted their concerns, specifically referencing the perceived detrimental impact of the 'Type A' contract on the continuity of care for their private patients ^99^. Leveraging their substantial bargaining power, these consultants engaged in lobbying efforts to voice their dissent. The influence of this concerted effort is evident in numerous media accounts ^93– 96, 99– 101^ and during the proceedings of the Special Committee on COVID-19 ^79^, where multiple TDs acknowledged being contacted by consultant constituents and private patients. Health officials also reflected on the pressure this created at the time, questioning the deal’s efficacy.

*‘I think it was quite an eye-opener, and the number of politicians that supported the consultants was quite surprising. […] We were under huge pressure, and Minister [for health] Harris was under huge pressure, particularly from consultants.’ – P1*.

*‘They [private hospital consultants] used every available channel, from direct approaches to the Department of Health and Taoiseach. […] I would say [consultants] shamelessly worked the patient angle and persuaded patients to advocate for them […] essentially about their terms and conditions.’ – P2*

These private consultants expressed dissatisfaction with the differential treatment between their counterparts in public hospitals, specifically those on ‘Type B’ and ‘Type C’ contracts who had not been *'stripped of their private practice rights’* (P5) as private practice was allowed to continue for contract holders with private practice rights in public hospitals and patients could continue to opt to become private patients in this context. However, this practice did not materialise, as all non-essential healthcare services were postponed. Consequently, all healthcare provided during this period was limited to essential, time-sensitive public health services.

*'What the consultants like me in the private sector needed was the Type 'C' contract. […] I never wanted the higher salary of Type 'A.' All I wanted was the ability to look after my existing patients to whom I have duty of care. […] that right was immediately removed from me. […] Our issue was that people [who] were employed by the HSE. You have not forced any contract change on them. But for us, the group of which needed the Type C contract. We were refused.' – P5.*

*‘They [Public Health Officials] basically said, right, either look after your patients in your private rooms and don't work in the hospital, or you work in the hospital, and you don't work in your rooms [off site consultation rooms]’ – P7.*

Hospital consultants who only work privately were also frustrated with the lack of involvement of their representative associations, the IMO, and the IHCA, in the SNA ^102^. Their distinct dissatisfaction manifested in establishing a new association in April 2020, the Medical Dental Consultant Association. With critical objectives to ‘ *Promote, encourage and support the advancement of the practice of private Medicine and Dentistry and to defend and protect the clinical independence of the members of the Association where necessary’* ^103^.

Public health officials admitted that one of the biggest challenges in negotiating and delivering the SNA was the active lobbying of private hospital consultants who exclusively practised in the private sector and were opposed to the deal. 

*‘The most difficult group for us to deal with was the pure private consultants.’ – P3.*

*‘The consultants yielded a great deal of power. That was evident in this arrangement without the consent or buy-in of the consultant body, you're going to find very difficult to push through any significant [health] reforms' – P2.*

The findings suggest that these challenges were further aggravated by the already fractured relationship and a lack of trust between public health officials and private hospital consultants. This was persistently evident in the interviews.

*‘How they [private hospital consultants] present things, everything is always presented as being in the best interests of their patients. […] The Department of Health and HSE have a more cynical view that the best interests and patients always seem to coincide with their own best interests. ' – P2.*

*‘There has been a complete disintegration [of the relationship] between the Department of Health and consultant bodies.’ – P5*

The interviews shed light on the considerable political influence wielded by medical consultants in Ireland. The role played by private hospital consultants exclusively working in the private sector emerged as a central challenge to the agreement. Their steadfast commitment to preserving 'clinical independence' was paramount, and they displayed a sense of unease regarding public sector involvement. Simultaneously, the public sector harboured its own grievances towards the private hospital consultants.

### Public administrative capacity to engage the private sector

These findings also present insights into the dynamics between Ireland's public and private health sectors. Specifically, they highlight the public sector's poor knowledge and understanding of the private health sector. One private sector participant believed this was evident in the terms and conditions of the Type A consultant contract.

*‘That contract was incredibly stupid. There was no understanding of how private practice operates whatsoever when that contract was implemented. They're a completely separate entity […] I'm a doctor, but I work for my medical company, the insurance companies pay my medical company, my medical company rents the clinic room that I'm operating in […] I don't think the public sector understands at all how private enterprise work.' – P7*

Public sector participants also reflected on their apparent misconception of private health care.

*‘It was probably not recognised fully the business model and that the private health care system is actually two blocks, which is the corporate bit then, the loose federation of private individuals who are not employees, and that got really complicated […] To change, private health care, and its role, that really needs to be understood, because you're actually changing a whole series of individual businesses of the private only consultants.’ – P5.*

However, public officials discussed the ambiguity surrounding the role of private healthcare in providing public health services, further complicating the situation. 

*'The state is unclear about what this mixed public-private system means. […] If there was more clarity, when we sat down to write out what to do, it would have been easier to say, well, these are the red lines. What we were doing the whole time was putting down a proposition to see what kind of an answer we would get. […] There isn't clarity about all the components of the current public-private mix and how that might evolve to a universal health care approach, and that became clear’ - P5.*

This view was also reflected by a lesson-learned survey carried out for the Department of Health/HSE in the aftermath of the SNA, which found that 70% of Hospital Group representatives and 60% of HSE staff felt their organisation’s role and objectives were not clearly defined in this agreement ^98^.

In the interviews, participants reflected that the experience of engaging with private sector operations, costs, and funding, which was previously uncharted territory for them, significantly influenced their perspectives on future health reform.

*‘It's not such a huge deal to take on the private system and make it available to everybody […] the government paid €100 million a month; the private system sustained itself on that €100 million a month, and we had cancer teams move from James’ and Tallaght up to the Beacon and cross to the Mater and all of that, worked.’ – P5.*

*‘What this shows is, it's [a single tier hospital system] possible for three months, it's deemed to be a good thing for three months when you're facing pandemics or severe, unknown, unquantifiable levels of illness. You're using it then to protect your sickest people. So, I think it does give learning for government’ – P3.*

Specifically, participants focused on achieving UHC in the Irish context, drawing on a UHI model.

*‘You could argue to insure the entire population and give them universal access to health care, low price, I'd said, that's one thing […] I think it's brought that discussion and potential question a bit more to the fore.’- P4.*

*‘What you can see quite quickly is that from a provider point of view, you could quite quickly set up a common trading platform that would repay all hospitals. […] It's possible to do a funding model [...] It's not a huge cost to the state […] The HSE spends about a billion a year on elective work, you could put that billion and the €2 billion from the private into one pot and say pay for all elective work and have an elective fund and let all hospitals bid into that, but have no differentiation in terms of the categorisation of patients into public and private.’ – P3*

The findings identify a complicated relationship between the public and private health sectors exacerbated by the absence of clear policy lines on private sector engagement. This, in turn, fuels a misunderstanding of the role of private practice in public healthcare delivery. Moreover, the experience of public health officials in negotiating and implementing the SNA contributed to enhanced knowledge and policy insights for developing and financing integrated public and private healthcare delivery systems to address existing capacity constraints in the public system. This experience has allowed policymakers and health system leaders to reconsider potential pathways for a UHI model, aiming to facilitate universal access to hospital care.

## Discussion

This research examines the policy process surrounding the SNA established between the HSE and Irish private hospitals in March 2020, serving as a case study within the Irish health system. Employing qualitative methods of documentary analysis and semi-structured interviews, the study comprehensively analysed the policy process, including the content of the SNA, its contextual foundations, and stakeholders' various experiences and roles. This investigation provides valuable insights into the influence of the medical profession and public administrative capacity to engage the private sector. It allows us to garner lessons on the governance of public-private relationships in the healthcare sector with the aim of achieving universal access to COVID care within an existing two-tier acute hospital system. Additionally, it enables us to draw possibilities worth considering for future health reform.

In response to the surge in COVID-19 cases in Europe, one of the initial measures taken by Government to combat the spread in Ireland was its inclusion on the list of notifiable infectious diseases specified under the 1947 Health Act ^80^. This legislation was initially championed by then Minister for Health, Dr Noel Browne, as part of his broader efforts to universalise health services for the Irish population and, at the time, was designed to curb the spread of TB during the late 1940s and early 1950s. It included provisions to exempt all patients suffering from a notifiable disease from statutory public hospital charges ^34, 40^. This legislation set the foundation for universal, equitable access to COVID-19 care in Ireland in 2020, including all public and private hospital provision.

The Irish government and key public health officials quickly recognised a significant challenge in implementing crucial health policy measures due to the insufficient and restricted capacity of public healthcare services. Rapidly scaling up and deploying essential new services are considerably more complex in a system lacking UHC principles ^10– 13, 104, 105^. This challenge became particularly evident when public hospitals faced the risk of being overwhelmed, ensuring that access to COVID-19 treatment was not contingent on financial means and eliminating scenarios where individuals could "skip the queue" – a reality in Ireland's two-tier hospital system ^45, 49, 50, 53, 62^. Ireland’s experience demonstrates the significance of agile responses and rapid implementation of health policy measures including universal access to hospital care through the SNA ^17, 18, 29, 63^. This move starkly contrasts the historical rigidity and siloed nature of public and health system bureaucracies and entrenched neoliberal policies in Ireland ^46, 54, 106, 107^.

As the SNA was agreed and rolled out, private hospital consultants effectively wielded political influence and leveraged their private patients to advocate for amendments to their terms and conditions. Public health officials perceived this strategy as a successful attempt by consultants to safeguard their independence and income, thereby shaping policies in their best interests. The negotiation processes surrounding consultant contracts highlight the enduring influence and gatekeeping role of the Irish medical profession, which employs its terms and conditions to impede transformative changes in the Irish healthcare domain ^39^. This obstruction is particularly evident in efforts to reform policies toward UHC and threaten private practice rights ^34, 40, 41, 47, 48^.

Insights from health systems and policy research highlight how poorly designed relationships with the private sector can weaken existing public structures, compromising the goals and desired outcomes ^19, 21, 108^. Health reform carries substantial ethical implications and necessitates political decisions that involve extensive negotiations with various interest groups. This process often alters the distribution of entitlements, responsibilities, and resources within the health system ^67, 109^. These elements are evident in this case study. Such changes can influence a diverse range of stakeholders with varying interests, power dynamics, and levels of influence, thereby creating both political opportunities and tensions ^67, 110^. Depending on the financial conditions, incentives, and intellectual influence related to their contributions, actors can impact the health policy process and reform ^67, 104, 109, 111^.

This research identified critical disjunctures between public and private actors in negotiating and delivering the SNA. The absence of clearly defined policies governing private sector engagement and the ambiguous understanding of the public-private mix in the Irish context hinder the health systems' ability to effectively execute agreements in the public interest. Establishing clear guidelines for interaction between the public and private sectors in non-crisis times is crucial for building trust and fostering robust partnerships and would help ensure better relationships and responses during times of crisis ^19, 21^. Currently, the private sector's commitment to aligning with public health goals is compromised by the lack of explicit medium and long-term policies and agendas ^112– 114^, complicating the realisation of shared objectives, including universal healthcare.

The evolving policy landscape of private sector utilisation during the COVID-19 pandemic highlights a significant transformation where an agreement becomes a complex facet of the health system's response to capacity challenges ^26, 27^, introducing dual policies on private sector engagement. The Irish public health system has been grappling with longstanding challenges for over two decades, exacerbated by difficulties post-pandemic and a May 2021 cyber-attack ^115– 117^. While these challenges are not unique to Ireland ^115, 118^, strategies to address backlogs, such as the National Treatment Purchase Fund (NTPF) and direct procurement by the HSE from private hospitals, reflect a fragmented policy approach. Recognising the effectiveness of one-off additional funding strategies, such as purchasing care from the private sector for short-term relief, is acknowledged that these approaches do not ensure sustained reductions in waiting times ^119^. Echoing this pattern, the NTPF initially reduced waiting numbers, but these improvements were unsustainable, resulting in persistent waiting times a decade later ^44^. This study emphasises the critical need for reassessing dual and overlapping strategies in private procurement within Ireland's public health system, highlighting the potential drift towards privatisation ^120^, thereby threatening the core objective of addressing capacity issues in public hospitals.

Finally, the SNA introduced new insights, leading public officials to contemplate consolidating public and private capacities for universal access to elective hospital care potentially through a UHI model. While a UHI model had been explored in 2011, it was abandoned in 2016 due to cost concerns ^55^. The SNA provides precise cost estimates for running the private hospital system and a comprehensive assessment of the system's total capacity—a previously elusive figure. Policymakers drew inferences from this, and considered re-evaluating pathways to a UHI model for hospital care in Ireland, aligning with insights explored by Thomas *et al.* in 2008 ^33, 121^.

## Conclusion

The emergence of the COVID-19 pandemic compelled the Irish government and policymakers to navigate unprecedented challenges, prioritising public health and equitable access to care amidst the impending crisis. However, this period of private hospital sector engagement also provided valuable insights into the public healthcare system, offering stakeholders critical knowledge and experience that could potentially guide achieving universal access to hospital care in Ireland post-COVID.

This research sheds light on the intricate policy process dynamics surrounding the negotiation and delivery of private hospital agreements in delivering universal hospital care during the COVID-19 pandemic in Ireland for a three-month period. It underscores the crucial role of broader socio-political contexts and the influence of various stakeholders - including government officials, policymakers, and the medical sector – and uncovers the complex and contested terrain within which struggles over UHC implementation during a crisis occur.

By exploring opportunities, challenges and dilemmas encountered when navigating the intersection of public and private healthcare in a dynamic socio-political context, this research offers important lessons for health system reform in Ireland and internationally. In particular, crucial insights are gleaned that potentially contribute to developing clear long-term goal alignment, robust governance practices, and trust-building mechanisms for successful public-private collaborations in two-tier health systems, informing future healthcare policy and implementation strategies.

## Ethical and security consideration

In this research article, data were derived from two primary sources: documents and interviews. The interview transcripts are not being uploaded to a repository, even in anonymised form, due to the sensitive nature of the interviews and the explicit guarantee of complete confidentiality and anonymity provided to the interviewees in their informed consent forms. Respecting the ethical commitments made to the interviewees and in alignment with the approval obtained from the Research Ethics Committee of the Centre for Health Policy and Management and Centre for Global Health in Trinity College Dublin’s School of Medicine in Trinity College Dublin, we cannot provide the interview transcripts. Any violation of these agreements could undermine the trust established with the research team, potentially causing harm to the integrity of the study.

It is important also to note that, given the relatively small size of those involved in this case study and in the policy community in Ireland, both historically and presently, revealing anonymised transcripts, may lead to revealing interviewees' identities by individuals working in health policy in the country. Such recognition could compromise the confidentiality promised to the participants and jeopardize the relationships fostered during the research process.

## Data Availability

Dataset one is made up of secondary, publicly available materials. Notably, all documents listed in the data inventory are now publicly accessible, accompanied by public links for easy reference. However, it's essential to highlight that two specific documents deviate from this norm. These two were shared with the researcher under conditions of confidentiality and trust by a participant in the research, and as such, they are not publicly linked to maintain the strict confidentiality agreement. Dataset two includes human data, which, although anonymised, is not publicly shared in order to protect the privacy of respondents in accordance with Trinity College Dublin guidance on Open Data and Research Data Management ( https://www.tcd.ie/library/riss/research-data.php). Our respondents may be identifiable given the size of the Irish health and social care community and the scope of the topics under discussion. Interview transcripts can be shared if requested by email to the corresponding author if the intended use is clearly explained and assured, the study is under the approval of a recognised ethics committee, and respondents’ privacy is explicitly assured under a formal agreement between all parties. Open Science Framework: Examining Universal Access to Acute Hospital Care in Ireland During the First Three Months of COVID-19: Lessons from the Policy Process. DOI: https://doi.org/10.17605/OSF.IO/T2GP5 ^122^. This project contains the following extended data: Supplementary file 1: Document Analysis Index Extended data file 1: Participant information leaflet Extended data file 2: Copy of the consent form Extended data file 3: Qualitative interview guide Data are available under the terms of the Creative Commons Attribution 4.0 International license (CC-BY 4.0).

## References

[1] Bambra C Riordan R Ford J : The COVID-19 pandemic and health inequalities. *J Epidemiol Community Health.* 2020;74(11):964–968. 10.1136/jech-2020-214401 32535550 PMC7298201

[2] Webb E Hernández-Quevedo C Williams G : Providing health services effectively during the first wave of COVID-19: A cross-country comparison on planning services, managing cases, and maintaining essential services. *Health Policy.* 2022;126(5):382–90. 10.1016/j.healthpol.2021.04.016 34246501 PMC8093167

[3] Winkelmann J Webb E Williams GA : European countries’ responses in ensuring sufficient physical infrastructure and workforce capacity during the first COVID-19 wave. *Health Policy.* 2022;126(5):362–72. 10.1016/j.healthpol.2021.06.015 34311982 PMC9187509

[4] Greer SL Rozenblum S Falkenbach M : Centralizing and decentralizing governance in the COVID-19 pandemic: The politics of credit and blame. *Health Policy.* 2022;126(5):408–17. 10.1016/j.healthpol.2022.03.004 35331575 PMC8913406

[5] Schmidt AE Merkur S Haindl A : Tackling the COVID-19 pandemic: Initial responses in 2020 in selected social health insurance countries in Europe. *Health Policy.* 2022;126(5):476–84. 10.1016/j.healthpol.2021.09.011 34627633 PMC9187505

[6] Toshkov D Carroll B Yesilkagit K : Government capacity, societal trust or party preferences: what accounts for the variety of national policy responses to the COVID-19 pandemic in Europe? *J Eur Public Policy.* 2022;29(7):1009–28. 10.1080/13501763.2021.1928270

[7] OECD: Health at a Glance: Europe 2020: State of Health in the EU Cycle. Paris: Organisation for Economic Co-operation and Development,2020; [cited 2023 Aug 11]. 10.1787/82129230-en

[8] Colbourn T : Unlocking UK COVID-19 policy. *Lancet Public Health.* 2020;5(7):e362–e363. 10.1016/S2468-2667(20)30135-3 32502388 PMC7266600

[9] Lal A Abdalla SM Chattu VK : Pandemic preparedness and response: exploring the role of universal health coverage within the global health security architecture. *Lancet Glob Health.* 2022;10(11):e1675–83. 10.1016/S2214-109X(22)00341-2 36179734 PMC9514836

[10] Ranabhat CL Jakovljevic M Kim CB : COVID-19 Pandemic: An Opportunity for Universal Health Coverage. *Front Public Health.* 2021;9: 673542. 10.3389/fpubh.2021.673542 34395361 PMC8358071

[11] Lal A Erondu NA Heymann DL : Fragmented health systems in COVID-19: rectifying the misalignment between global health security and universal health coverage. *Lancet.* 2021;397(10268):61–7. 10.1016/S0140-6736(20)32228-5 33275906 PMC7834479

[12] Galvani AP Parpia AS Pandey A : Universal healthcare as pandemic preparedness: The lives and costs that could have been saved during the COVID-19 pandemic. *Proc Natl Acad Sci U S A.* 2022;119(25): e2200536119. 10.1073/pnas.2200536119 35696578 PMC9231482

[13] Thomas S Sagan A Larkin J : Strengthening health systems resilience: key concepts and strategies. World Health Organization. Regional Office for Europe,2020;29. 32716618

[14] WHO: Sustainable health financing, universal coverage and social health insurance. World Health Organization; Report No.: WHA58.33.2005; [cited 2023 May 11]. Reference Source

[15] Montserrat D : Report on the COVID-19 pandemic: lessons learned and recommendations for the future.2023; [cited 2023 Sep 14]. Reference Source

[16] Rajan S McKee M Hernández-Quevedo C : What have European countries done to prevent the spread of COVID-19? Lessons from the COVID-19 Health system response monitor. *Health Policy.* 2022;126(5):355–61. 10.1016/j.healthpol.2022.03.005 35339282 PMC8912990

[17] Burke S Parker S Fleming P : Building health system resilience through policy development in response to COVID-19 in Ireland: From shock to reform. *Lancet Reg Health Eur.* 2021;9: 100223. 10.1016/j.lanepe.2021.100223 34642676 PMC8495249

[18] Kennelly B O’Callaghan M Coughlan D : The COVID-19 pandemic in Ireland: An overview of the health service and economic policy response. *Health Policy Technol.* 2020;9(4):419–29. 10.1016/j.hlpt.2020.08.021 32923355 PMC7480279

[19] Maresso A Waitzberg R Tille F : Engaging the private sector in delivering health care and goods: governance lessons from the COVID-19 pandemic. European Observatory on Health Systems and Policies; Report No.: 56.2023;34. Reference Source 39541487

[20] World Health Organization: Engaging the private health service delivery sector through governance in mixed health systems: strategy report of the WHO Advisory Group on the Governance of the Private Sector for Universal Health Coverage. Geneva: World Health Organization;2020; [cited 2023 Nov 16]. Reference Source

[21] Clarke D Doerr S Hunter M : The private sector and universal health coverage. *Bull World Health Organ.* 2019;97(6):434–5. 10.2471/BLT.18.225540 31210681 PMC6560377

[22] Duckett S : Commentary: The Consequences of Private Involvement in Healthcare - The Australian Experience. *Healthc Policy.* 2020;15(4):21–5. 10.12927/hcpol.2020.26228 32538345 PMC7294448

[23] Heavey P : The Irish Healthcare System: A Morality Tale. *Camb Q Healthc Ethics.* 2019;28(2):276–302. 10.1017/S0963180119000100 31113514

[24] Greer SL Méndez CA : Universal Health Coverage: A Political Struggle and Governance Challenge. *Am J Public Health.* 2015;105 Suppl 5(Suppl 5):S637–9. 10.2105/AJPH.2015.302733 26180991 PMC4627521

[25] Fox AM Reich MR : The Politics of Universal Health Coverage in Low- and Middle-Income Countries: A Framework for Evaluation and Action. *J Health Polit Policy Law.* 2015;40(5):1023–60. 10.1215/03616878-3161198 26195606

[26] Waitzberg R Hernández-Quevedo C Bernal-Delgado E : Early health system responses to the COVID-19 pandemic in Mediterranean countries: A tale of successes and challenges. *Health Policy.* 2022;126(5):465–75. 10.1016/j.healthpol.2021.10.007 34711444 PMC8507573

[27] van Ginneken E Reed S Siciliani L : Addressing backlogs and managing waiting lists during and beyond the COVID-19 pandemic. Copenhagen (Denmark): European Observatory on Health Systems and Policies; (European Observatory Policy Briefs),2022; [cited 2023 Nov 16]. Reference Source 36800878

[28] O’leary N Kingston L Griffin A : COVID-19 healthcare policies in Ireland: A rapid review of the initial pandemic response. *Scand J Public Health.* 2021;49(7):713–20. 10.1177/14034948211008371 34011221 PMC8521351

[29] Parker S Mac Conghail L Siersbaek R : How to not revert to type: Complexity-informed learnings from the pandemic response for health system reform and universal access to integrated care. *Frontiers in Public Health.* 2023;11: 1088728. 10.3389/fpubh.2023.1088728 36908402 PMC9996344

[30] Government of Ireland: Ireland’s National Action Plan in response to COVID-19 (Coronavirus).2020; [cited 2023 Aug 10]. Reference Source

[31] Central Statistics Office: System of Health Accounts 2019.CSO, Financing Schemes of Health Care Services,2019; [cited 2023 Nov 30]. Reference Source

[32] OECD: Ireland: Country Health Profile 2021, State of Health in the EU.Paris/European Observatory on Health Systems and Policies, Brussels.: OECD Publishing;2021; [cited 2023 Feb 2]. Reference Source

[33] Burke S : Reform of the Irish Healthcare System: What Reform?In: Murphy MP, Dukelow F, editors, *The Irish Welfare State in the Twenty-First Century: Challenges and Change*. London: Palgrave Macmillan UK;2016;167–91. 10.1057/978-1-137-57138-0_8

[34] Wren MA Connolly S : A European late starter: lessons from the history of reform in Irish health care. *Health Econ Policy Law.* 2019;14(3):355–73. 10.1017/S1744133117000275 29277162

[35] Mercille J : The Public–Private Mix in Primary Care Development: The Case of Ireland. *Int J Health Serv.* 2019;49(3):412–30. 10.1177/0020731419836079 30894048

[36] Day C : Report of the Independent Review Group established to examine the role of voluntary organisations in publicly funded health and personal social services. Department of Health.2019; [cited 2023 Sep 7]. Reference Source

[37] Central Statistics Office: Ireland’s UN SDGs 2019 - Report on Indicators for Goal 3 Good Health and Well-Being.CSO; Coverage of Essential Health Services,2019; [cited 2023 Nov 30]. Reference Source

[38] McGlacken-Byrne D Parker S Burke S : Tracking aspects of healthcare activity during the first nine months of COVID-19 in Ireland: a secondary analysis of publicly available data [version 2; peer review: 2 approved with reservations]. *HRB Open Res.* 2023;4:98. 10.12688/hrbopenres.13372.2 PMC11439124 39347503

[39] Malone P Millar M : “The only equality is the pain”: An exploration of the Irish policy sphere’s approach to “access” and “entitlement” in health care. *Soc Policy Adm.* 2020;54(1):163–177. 10.1111/spol.12531

[40] Barrington R : Health, Medicine and Politics in Ireland 1900-1970.Dublin: Institute of Public Administration,1987. Reference Source

[41] Wren MA : Unhealthy State: Anatomy of a Sick Society.New Island,2003;458. Reference Source

[42] van Olmen J Marchal B Van Damme W : Health systems frameworks in their political context: framing divergent agendas. *BMC Public Health.* 2012;12(1): 774. 10.1186/1471-2458-12-774 22971107 PMC3549286

[43] Mercille J : Privatization in the Irish hospital sector since 1980. *J Public Health (Oxf).* 2018;40(4):863–70. 10.1093/pubmed/fdy027 29462359

[44] Burke S Brugha R Thomas S : The National Treatment Purchase Fund – A success for some patients yet a public policy failure? *Administration.* 2019;67(2):47–69. 10.2478/admin-2019-0013

[45] Burke S Thomas S Barry S : Indicators of health system coverage and activity in Ireland during the economic crisis 2008-2014 - from 'more with less' to 'less with less'. *Health Policy.* 2014;117(3):275–8. 10.1016/j.healthpol.2014.07.001 25082466

[46] Fraser A Murphy E Kelly S : Deepening Neoliberalism via Austerity and ‘Reform’: The Case of Ireland. *Hum Geogr.* 2013;6(2):38–53. 10.1177/194277861300600204

[47] Burke S : Irish Apartheid: Healthcare Inequality in Ireland.Dublin: New Island;2009. Reference Source

[48] Wren MA Tussing AD : How Ireland Cares: The Case for Health Care Reform.Dublin: New Island;2006;434. Reference Source

[49] Burke S Barry S Siersbaek R : Sláintecare – A ten-year plan to achieve universal healthcare in Ireland. *Health Policy.* 2018;122(12):1278–1282. 10.1016/j.healthpol.2018.05.006 29843901

[50] Connolly S Wren MA : Universal Health Care in Ireland—What Are the Prospects for Reform? *Health Syst Reform.* 2019;5(2):94–9. 10.1080/23288604.2018.1551700 30875264

[51] The Health Insurance Authority: Annual reports & accounts.2019 Annual Report & Accounts,2020. Reference Source

[52] Duffy K Connolly S Maître B : Unequal chances? Inequalities in mortality in Ireland.ESRI; Sep,2022; [cited 2023 Aug 4]. Reference Source

[53] Connolly S Wren MA : Unmet healthcare needs in Ireland: Analysis using the EU-SILC survey. *Health Policy.* 2017;121(4):434–41. 10.1016/j.healthpol.2017.02.009 28233599

[54] Kirby P Murphy M : Towards a Second Republic: Irish Politics and the Celtic Tiger.Pluto Press;2011;240. 10.2307/j.ctt183p6s9

[55] Wren MA Connolly S Cunningham N : An Examination of the Potential Costs of Universal Health Insurance in Ireland.ESRI; Nov,2015; [cited 2023 Nov 16]. Reference Source

[56] Department of Health: The Path to Universal Healthcare: White Paper on Universal Health Insurance.Dublin: Department of Health,2014. Reference Source

[57] Darker CD Donnelly-Swift E Whiston L : Demographic factors and attitudes that influence the support of the general public for the introduction of universal healthcare in Ireland: A national survey. *Health Policy.* 2018;122(2):147–56. 10.1016/j.healthpol.2017.11.009 29198852

[58] Leahy P : Irish Times Poll: Health and housing most important issues for voters. *The Irish Times* . Feb,2020; [cited 2023 Apr 30]. Reference Source

[59] Committee on the Future of Healthcare: Sláintecare Repot.Dublin: Houses of the Oireachtas,2017. Reference Source

[60] de Buitléir D : Report of the Independent Review Group established to examine Private Activity in Public Hospitals.The Independent Review Group Established to Examine Private Activity in Public Hospitals,2019;145. Reference Source

[61] Thomas S Johnston B Barry S : Sláintecare implementation status in 2020: Limited progress with entitlement expansion. *Health Policy.* 2021;125(3):277–83. 10.1016/j.healthpol.2021.01.009 33531170 PMC9757858

[62] Connolly S Wren MA Keegan C : Towards universal healthcare in Ireland - what can we learn from the literature?ESRI; Sep,2023; [cited 2023 Oct 24]. 10.26504/sustat121

[63] Mercille J Turner B Lucey DS : Ireland’s takeover of private hospitals during the COVID-19 pandemic. *Health Econ Policy Law.* 2022;17(2):232–7. 10.1017/S1744133121000189 34001297 PMC8167255

[64] Barry S Stach M Thomas S : Understanding service reorganisation in the Irish health & social care system from 1998 to 2020: lessons for reform and transformation [version 1; peer review: 1 approved with reservations]. *HRB Open Res.* 2021;4:106. 10.12688/hrbopenres.13342.1

[65] Burke S Thomas S Stach M : Health system foundations for Sláintecare implementation in 2020 and beyond - co-producing a Sláintecare Living Implementation Framework with Evaluation: Learning from the Irish health system’s response to COVID-19. A mixed-methods study protocol [version 1; peer review: 2 approved]. *HRB Open Res.* 2020;3:70. 10.12688/hrbopenres.13150.1 33728398 PMC7934093

[66] Johnston BM Burke S Kavanagh PM : Moving beyond formulae: a review of international population-based resource allocation policy and implications for Ireland in an era of healthcare reform [version 1; peer review: 1 approved, 1 approved with reservations]. *HRB Open Res.* 2021;4:121. 10.12688/hrbopenres.13453.1

[67] Gilson L Orgill M Shroff ZC : A health policy analysis reader: the politics of policy change in low- and middle-income countries. World Health Organization;2018;143. Reference Source

[68] Silverman D : Doing Qualitative Research. SAGE;2010;474. Reference Source

[69] Walt G Gilson L : Reforming the health sector in developing countries: the central role of policy analysis. *Health Policy Plan.* 1994;9(4):353–70. 10.1093/heapol/9.4.353 10139469

[70] Walt G Shiffman J Schneider H : ‘Doing’ health policy analysis: methodological and conceptual reflections and challenges. *Health Policy Plan.* 2008;23(5):308–17. 10.1093/heapol/czn024 18701552 PMC2515406

[71] Gilson L Raphaely N : The terrain of health policy analysis in low and middle income countries: a review of published literature 1994-2007. *Health Policy Plan.* 2008;23(5):294–307. 10.1093/heapol/czn019 18650209 PMC2515407

[72] Sheikh K Gilson L Agyepong IA : Building the Field of Health Policy and Systems Research: Framing the Questions. *PLoS Med.* 2011;8(8): e1001073. 10.1371/journal.pmed.1001073 21857809 PMC3156683

[73] Yin RK : Case Study Research: Design and Methods.Thousand Oaks, CA: SAGE Publications;2009; [cited 2023 Jun 30]. Reference Source

[74] Bowen GA : Document Analysis as a Qualitative Research Method. *Qual Res J.* 2009;9(2):27–40. 10.3316/QRJ0902027

[75] Braun V Clarke V : Using thematic analysis in psychology. *Qual Res Psychol.* 2006;3(2):77–101. 10.1191/1478088706qp063oa

[76] Robson J Michels N Dawes Farquhar J : Triangulation in industrial qualitative case study research: Widening the scope. *Ind Mark Manag.* 2020;87:160–170. 10.1016/j.indmarman.2020.02.001

[77] Saldana J : The Coding Manual for Qualitative Researchers. 4th ed. SAGE Publications;2021;440. Reference Source

[78] OECD: Health at a Glance 2021: OECD Indicators. OECD; (Health at a Glance),2021; [cited 2023 Jan 31]. 10.1787/19991312

[79] Oireachtas H of the : Special Committee on Covid-19 Response debate - Use of Private Hospitals.2020; [cited 2023 Aug 9]. Reference Source

[80] Electronic Irish Statute Book: S.I. No. 53/2020 - Infectious Diseases (Amendment) Regulations 2020. Office of the Attorney General;2020; [cited 2023 Aug 10]. Reference Source

[81] Horgan-Jones J O’Connell H : Pandemonium: Power, Politics and Ireland’s Pandemic.Dublin: Gill Books;2022. Reference Source

[82] MerrionStreet.ie: Statement by An Taoiseach Leo Varadkar On measures to tackle Covid-19 Washington, 12 March 2020. 2020; [cited 2023 Aug 10]. Reference Source

[83] HSE: Framework for Pandemic Response with Private Hospitals.HSE;2020.

[84] HSE, Department of Health: Heads of Terms. Containing the basis of agreement in relation to the provision of public health services at private hospitals in response to the COVID-19 Pandemic. 2020. Reference Source

[85] HSE: Consultants Contract Common Purpose. 2020. Reference Source

[86] Irish Medical Council: Temporary Consultant Contract Change.[cited 2023 Aug 10]. Reference Source

[87] Collins D : Health Workforce Consultant Pay and Skills Mix, 2012-2017. Irish Government Economic and Evaluation Service, Department of Public Expenditure and Reform,2019; [cited 2023 Nov 30]. Reference Source

[88] HSE: Minutes of Special HSE Board Meeting Ref: COVID-19. 2020. Reference Source

[89] Department of Health: Email to the Private Secretary to the Minister for Health from Tony Holohan. 2020. Reference Source

[90] Crowley P Hughes A : The impact of the COVID-19 pandemic and the societal restrictions on the health and wellbeing of the population, on our staff and on health service capacity and delivery: A plan for healthcare and population health recovery. Dublin: National QI Team, HSE;2021. Reference Source

[91] Corona Citizens Science Project: Corona Citizens Science Project - Wave 2 Results. 2020; [cited 2023 Aug 10]. Reference Source

[92] HSE: Minutes of Special HSE Board Meeting Ref: COVID-19.2020; [cited 2023 Nov 12]. Reference Source

[93] Wall M : Covid-19: Controversial private hospitals €340m deal leaves legacy of angry doctors. *The Irish Times.* 2020; [cited 2023 Aug 10]. Reference Source

[94] RTÉ: HSE deal with private hospitals “has to stop now.”. May 15,2020; [cited 2023 Aug 10]. Reference Source

[95] Ryan O : Consultant says private hospital contract is ‘a bad deal’ for taxpayers. *TheJournal.ie. * 2020; [cited 2023 Aug 10]. Reference Source

[96] Wall M : Doctors claim they have been forced to cancel private hospital procedures. *The Irish Times. * 2020; [cited 2023 Aug 10]. Reference Source

[97] HSE. Minutes of Special HSE Board Meeting Ref COVID-19.2020; [cited 2023 Nov 10]. Reference Source

[98] Prospectus: HSE Private Hospital Partnership. Lessons Learned Review. Dublin;2020.

[99] Wall M : Private hospital consultants urged not to sign up to working in public system. *The Irish Times*.2020; [cited 2023 Aug 10]. Reference Source

[100] Wall M : Coronavirus: Plans to move 600 consultants into public system hit obstacle. *The Irish Times. * 2020; [cited 2023 Aug 10]. Reference Source.

[101] Fagan M : Private doctors warn proposed Covid contract will create new public waiting list problem. *Irish Examiner*.2020; [cited 2023 Aug 10]. Reference Source

[102] Cahill N : Animosity within profession over private hospital deal. *Medical Independent*.2020; [cited 2023 Aug 10]. Reference Source

[103] MDCA. Medical & Dental Consultants Association:2023; [cited 2023 Aug 10]. Reference Source

[104] Kutzin J Sparkes SP : Health systems strengthening, universal health coverage, health security and resilience. *Bull World Health Organ.* 2016;94(1):2. 10.2471/BLT.15.165050 26769987 PMC4709803

[105] Unruh L Allin S Marchildon G : A comparison of 2020 health policy responses to the COVID-19 pandemic in Canada, Ireland, the United Kingdom and the United States of America. *Health Policy.* 2022;126(5):427–37. 10.1016/j.healthpol.2021.06.012 34497031 PMC9187506

[106] Janssen M van der Voort H : Agile and adaptive governance in crisis response: Lessons from the COVID-19 pandemic. *Int J Inf Manage.* 2020;55: 102180. 10.1016/j.ijinfomgt.2020.102180 32836637 PMC7309933

[107] Peterson CL Walker C : Universal health care and political economy, neoliberalism and effects of COVID-19: A view of systems and complexity. *J Eval Clin Pract.* 2022;28(2):338–40. 10.1111/jep.13631 34647671 PMC8656626

[108] Vecchi V Casalini F Cusumano N : PPP in Health Care—Trending Toward a Light Model: Evidence From Italy. *Public Works Management & Policy.* 2020;25(3):244–58. 10.1177/1087724X20913297

[109] Sparkes SP Bump JB Özçelik EA : Political Economy Analysis for Health Financing Reform. *Health Syst Reform.* 2019;5(3):183–94. 10.1080/23288604.2019.1633874 31369319

[110] Erasmus E Gilson L : How to start thinking about investigating power in the organizational settings of policy implementation. *Health Policy Plan.* 2008;23(5):361–8. 10.1093/heapol/czn021 18664526

[111] Lipsky M : Street Level Bureaucracy: Dilemmas of the Individual in Public Services.Russell Sage Foundation,1980; [cited 2023 Jul 2]. Reference Source

[112] Cionnaith FÓ : Private hospitals “need to do right thing” - Donnelly.RTÉ,2023; [cited 2023 Nov 16]. Reference Source

[113] McAuley E : HSE CEO “doesn’t accept” he was too late in approach to private hospitals on winter beds.TheJournal.ie.2023; [cited 2023 Nov 16]. Reference Source

[114] McNally T : “Extremely busy” private hospitals cannot support public health system “ad hoc.”. Irish Examiner.2023; [cited 2023 Nov 16]. Reference Source

[115] Department of Health: Waiting List Action Plan 2022. 2022; [cited 2023 Nov 16]. Reference Source

[116] HSE: Performance Profile April to June 2022. 2022; [cited 2022 Dec 4]. Reference Source

[117] HSE: Winter Plan October 2022 – March 2023.Health Service Executive (HSE);,2022; [cited 2023 Feb 2]. Reference Source

[118] Brick A Keegan C : Paying more to wait less: Estimating the cost of reducing Ireland’s public hospital waiting lists.ESRI;2020; [cited 2023 Nov 16]. Reference Source

[119] Siciliani L Moran V Borowitz M : WHAT WORKS? WAITING TIME POLICIES IN THE HEALTH SECTOR. 2015. Reference Source

[120] Cullen P : HSE board members warn of ‘drift towards privatisation’ in efforts to cut waiting lists. *The Irish Times*.2023; [cited 2023 Nov 5]. Reference Source

[121] Thomas S Normand C Smith S : Social health insurance: further options for Ireland. 2008; [cited 2023 Nov 5]. Reference Source

[122] Mac Conghail LM : Examining Universal Access to Acute Hospital Care in Ireland During the First Three Months of COVID-19: Lessons from the Policy Process. 2024; [cited 2024 Jan 18]. 10.17605/OSF.IO/T2GP5 PMC11803191 39927194

