# Supplementary material for: Examining universal access to acute hospital care in Ireland during the first three months of COVID-19: Lessons from the policy process
Source: HRB Open Res. 2024 Feb 15;7:4. [Version 1] doi: 10.12688/hrbopenres.13848.1 (PMC11803191; doi:10.12688/hrbopenres.13848.1)
Supplement: Peer review history

# Reviewer report: Reviewer response for version 1

**DOI:** 10.21956/hrbopenres.15174.r43609

**Published:** 2024-12-18

**Authors:** Arkalgud Ramaprasad

**Affiliations:** 1 University of Illinois at Chicago, Chicago, IL, USA

The paper addresses the important issue of partnership between private and public hospitals for the delivery of acute care, especially in the context of a pandemic like Covid-19. It is a rich descriptive study with some explanatory power. Its findings may have limited power to predict and regulate the behavior of hospitals in future crises. It is a grounded qualitative study. The researchers adhere to the requirements of the method.

 The major limitation of the study and its generalizability is the absence of a logical framework. There does not appear to be such a framework to guide the collection of data and its analysis. Nor do the authors present a framework for future application based on their study. The absence of a framework results in the errors of omission in data collection and analysis inherent in grounded research.

 I shall explain my comment using the ontology of the problem I have constructed based on the paper. The ontology may be accessed via the link in this comment since the format of the review input does not allow the insertion of an image.

https://hrbopenresearch.s3.eu-west-1.amazonaws.com/supplementary/13848/105c527c-c08c-4f2a-ad8c-40313a3d7f06.pdf

**Figure: Ontology of Policies for Universal Access to Acute Hospital Care**

 The ontology reorganizes the key words/phrases in the paper as Outcomes, Objectives, Hospital type, Impetus, and Actors. The actors provide the impetus to the hospitals to seek the objectives for the desired outcomes of acute care. The desired outcomes are accessible, quality, affordable, and equitable acute care. To deliver such care, the private and public hospitals must align their goals, modify governance, and establish trust. The government officials, healthcare managers, professional associations, and healthcare professionals are the actors who provide the impetus to achieve the objectives and consequently the outcomes. The impetus may enable the objectives, normalize them, or impede them.  The key words/phrases organized as an ontology encapsulate 4*3*2*3*4 = 288 pathways for universal access to acute hospital care.

 Seen through the lens of the ontology as a framework, many errors of omission in data collection and analysis become clear. For example, the dominant focus of the paper is on enablers. While issues of normalization (for example, of remuneration) and impediments (for example, to change in governance) are mentioned, their inclusion does not appear to be systematic included in data collection and analysis. It is not clear whether the actors interviewed were systematically asked about all three impetuses, or only the enablers? Similarly, were all three sought in the analysis of the documents. The transcripts of the respondent comments clearly show all three impetuses.

 Similarly, the transcripts quoted in the paper highlight a rich interaction between the actors, their impetuses, and their potential effects on the hospitals’ objectives and the acute care outcomes. They are presented anecdotally; they could be mapped systematically onto a framework such as the ontology to obtain deeper insights into the challenges of private-public partnerships among hospitals for acute care in a crisis. Such a mapping may highlight pathways that were effective and may be reinforced, the ones that are ineffective and must be redirected, and the ones that were not explored and could be in the future.

 In summary, the paper is good but has a richer potential than has been presented.

Is the work clearly and accurately presented and does it cite the current literature?

Yes

If applicable, is the statistical analysis and its interpretation appropriate?

Yes

Are all the source data underlying the results available to ensure full reproducibility?

Yes

Is the study design appropriate and is the work technically sound?

Partly

Are the conclusions drawn adequately supported by the results?

Yes

Are sufficient details of methods and analysis provided to allow replication by others?

Yes

Reviewer Expertise:

Policy design in healthcare and other areas

I confirm that I have read this submission and believe that I have an appropriate level of expertise to confirm that it is of an acceptable scientific standard.

# Reviewer report: Reviewer response for version 1

**DOI:** 10.21956/hrbopenres.15174.r38429

**Published:** 2024-03-23

**Authors:** Brian Turner

**Affiliations:** 1 University College Cork, Cork,, Ireland

**General Comments**

 This is a very interesting and timely insight into what was an emergency measure in the early days of the pandemic, but from which learnings can be taken for future health policy.  The researchers are to be commended for undertaking this study.

**Specific Comments**

 Page 4, Paragraph 1 – The eligibility criteria for GP Visit cards has now been expanded to include children under the age of eight (up from six).

 Page 4, Paragraph 3 – There are more up-to-date figures for health insurance take-up, the latest being from Q3 2023, when the proportion of the population with cover, based on the updated population figures from the 2022 Census, was 46.8% (see Market reports and bulletins | The Health Insurance Authority (hia.ie)).

 Page 6, Paragraph 4 – The two references cited after the number of beds and occupancy rates are dated 1987 and 2003.  Presumably the figures given were taken from more recent publications, which should also be cited here.

 Page 9, Paragraph 15 – The researchers note that “participants focused on achieving UHC in the Irish context, drawing on a UHI model.”  Did the idea of the UHI model come independently from the participants or was it suggested by the researchers?

 Page 10, Paragraph 6 – The authors refer here to “shared objectives, including universal healthcare”.  Is there any evidence that this is a shared objective of both the public and private sectors in Ireland?  Or are the authors specifically referring to the pandemic scenario rather than a general ideological objective of universal healthcare?

 Page 11, Paragraph 1 – I would urge caution about potentially overstating the precision of cost estimates for running the private hospital system arising from the SNA, as this was not indicative of the ‘normal’ functioning of the system.

**Minor Comments**

 Page 3, paragraph 8 – The third line should read means testing (rather than mean testing).

Is the work clearly and accurately presented and does it cite the current literature?

Yes

If applicable, is the statistical analysis and its interpretation appropriate?

Not applicable

Are all the source data underlying the results available to ensure full reproducibility?

Yes

Is the study design appropriate and is the work technically sound?

Yes

Are the conclusions drawn adequately supported by the results?

Yes

Are sufficient details of methods and analysis provided to allow replication by others?

Yes

Reviewer Expertise:

Health economics, health funding, Irish health system

I confirm that I have read this submission and believe that I have an appropriate level of expertise to confirm that it is of an acceptable scientific standard.
